# Characterization of bone repair in rat femur after treatment with calcium phosphate cement and autogenous bone graft

**DOI:** 10.1186/1746-160X-6-10

**Published:** 2010-06-28

**Authors:** Edela Puricelli, Adriana Corsetti, Deise Ponzoni, Gustavo L Martins, Mauro G Leite, Luis A Santos

**Affiliations:** 1 Oral and Maxillofacial Surgery Unit, Hospital de Clinicas de P.A., School of Dentistry, UFRGS, Porto Alegre, RS, Brazil; 2 Universidade Federal do Rio Grande do Sul, Porto Alegre, RS, Brazil; 3 School of Dentistry, Universidade Federal do Rio Grande do Sul, Porto Alegre, RS, Brazil; 4 Universidade Federal de Pelotas, Pelotas, RS, Brazil; 5 The School of Engineering of Materials and School of Dentistry, Universidade Federal do Rio Grande do Sul, Porto Alegre, RS, Brazil

## Abstract

**Background:**

In this study, the biocompatibility, stability and osteotransductivity of a new cement based on alpha-tricalcium phosphate (alpha-TCP) were investigated in a bone repair model using a rat model.

**Methods:**

The potential of alpha-TCP on bone repair was compared to autogenous bone grafting, and unfilled cavities were used as negative control. Surgical cavities were prepared and designated as test (T), implanted with alpha-TCP blocks; negative control (C - ), unfilled; and positive control (C + ), implanted with autogenous bone graft. Results were analyzed on postoperative days three, seven, 14, 21 and 60.

**Results:**

The histological analyses showed the following results. Postoperative day three: presence of inflammatory infiltrate, erythrocytes and proliferating fibroblasts in T, C - and C + samples. Day seven: extensive bone neoformation in groups T and C + , and beginning of alpha-TCP resorption by phagocytic cells. Days 14 and 21: osteoblastic activity in the three types of cavities. Day 60: In all samples, neoformed bone similar to surrounding bone. Moderate interruption on the ostectomized cortical bone.

**Conclusions:**

Bone neoformation is seen seven days after implantation of alpha-TCP and autogenous bone. Comparison of C - with T and C + samples showed that repair is faster in implanted cavities; on day 60, control groups presented almost complete bone repair. Alpha-TCP cement presents biocompatibility and osteotransductivity, besides stability, but 60 days after surgery the cavities were not closed.

## Background

Bone exists in two main structural types: primary bone and lamellar or secondary bone [1]. Bone repair occurs in a process that may take months or years [2]. The morphological and functional recovery of hard tissues lost during the treatment of pathological processes and traumatic lesions has been extensively studied, and different approaches have been suggested.

Autogenous bone graft is considered to be the gold standard for replacement of lost tissue [3,4]. Garg, in 1999 [5], defined the three processes associated to the fate of bone grafts: osteogenesis, osteoinduction and osteoconduction. Osteogenesis is formation of bone, whereas osteoinduction is the process by which osteogenesis is induced and osteoconduction is a physiologic process whereby a conductor provides a physical matrix for deposition of new bone tissue. Advancements in surgical techniques to collect human bone for autogenous grafting are not able to keep pace with the evolution in the production of alloplastic material, such as calcium phosphate cements [6], which have been successfully used for bone repair in the last decade.

The ideal material must be biocompatible, bioactive and resorbable [7]. Other desirable characteristics include unlimited availability, stability and ability of filling and conformation [8]. Scaffold design is of primordial importance for the success of bone tissue-engineering grafts, and a wide variety of biomaterials, including polymers, ceramics and composites) are under investigation for bone repair (reviewed by Fröhlich et al. [9]). The association of biomaterials with stem/progenitor cells [10] or their use as vehicles for cytokines, growth factors or genes for bone formation [11] represent important additions to the field of regenerative medicine. Presently, however, no single biomaterial available for bone repair and regeneration presents all the properties required for an ideal bone graft (reviewed by [12]), and new combinations of materials are under intensive research.

Brown and Chow (1986) [13] were the first to propose the use of calcium phosphate cement in bone repair. Its biocompatibility, bioactivity and osteoconductivity have been shown in many studies [14-17], and its biological behavior has been investigated *in vivo *[6,18]. In general, these cements are absorbed by the intense activity of the phagocytic system, leading to the simultaneous formation of new bone tissue in the interface bone/implant. This process is called osteotransductivity [19]. Knabe et al. [20] suggested that it is a slow process, which continues in average for two years after implantation. Toquet et al. [21] analyzed the osteogenic potential of human bone marrow cells during *in vitro *culture on calcium phosphate ceramics, showing that the cells populated the pores of the material.

Santos, in 2002 [22], developed a new cement based on alpha-tricalcium phosphate [Ca _3_(PO _4_) _2_] (alpha-TCP) by adding a fluid reducer, ammonium polyacrylate, to this material. This new type of calcium phosphate showed greater resistance to mechanical stress while maintaining the characteristics of osteotransductivity and biocompatibility. Considering the therapeutic potential of calcium phosphate cements, the present work aimed to contribute to this area of research by the histological analyses of the effect of a new type of biomaterial, alpha-tricalcium phosphate cement, as compared to autogenous bone grafting, during bone repair in surgically created cavities. Characteristics of alpha-TCP, such as biocompatibility, stability and osteotransductivity were also investigated.

The study was conducted in a rat model, in which surgical cavities were created according to the protocol established by Puricelli [23,24]. The bone cavity has only one ruptured cortical margin, allowing a type of fixation of the bone fragments as an essential condition for the production of a bone callus. Although more frequently critical-sized bone defects of 5 mm are created to assess healing progress in rats, smaller defects are also used. Cao and Kuboyama [25], for instance, compared the therapeutic potential of scaffolds composed of polyglycolic acid and beta-tricalcium phosphate (PGA/β-TCP) or hydroxylapatite, in a rat model in which 3 mm × 2 mm femur defects were made. Studies conducted by our group [19,20,26,27] have also shown that 2 mm × 4 mm cavities, surgically induced on the cortical surface of the femur, represent an adequate model to investigate the role of different materials and processes on bone healing.

## Methods

This controlled experimental study was conducted in the Laboratory of Experimental Surgery of the Oral and Maxillofacial Surgery and Traumatology Discipline, School of Dentistry of Universidade Federal do Rio Grande do Sul (UFRGS). The biomaterial used was produced in the Department of Materials of the School of Mechanical Engineering UFRGS. Thirty precured, cylindrical blocks of alpha-tricalcium phosphate (alpha-TCP) with 2 mm diameter and 4 mm length were produced. The material was sterilized in hydrogen peroxide, following methods established at Hospital de Clínicas de Porto Alegre (HCPA, 2002) and Corsetti et al. [28].

Thirty 5-month old male Wistar rats were used, an age at which sexual/social maturity is reached. The animals were housed and maintained in accordance with the guidelines for the care of laboratory animals, Normative Resolution 04/97, prepared by the Ethics and Health Research Committee/GPPG/HCPA. The project was approved by the Research Ethics Committee of the School of Dentistry of Universidade Federal do Rio Grande do Sul.

The animals were anesthetized and the right hind leg was shaved and the skin disinfected. A 3 cm incision was made on the skin, the tissues were separated by layers and the periosteum was incised with a scalpel. Surgical cavities were prepared on the cortical surface of the femur with help of a perforated titanium surgical splint. Three cavities were produced with a slow-rotation trephine bur, under constant irrigation with physiological saline solution and aspiration. The cavities, measuring 2 mm wide and 4 mm deep, were designated as test (T), negative control (C - ) and positive control (C + ), from proximal to distal.

**Figure 1:**
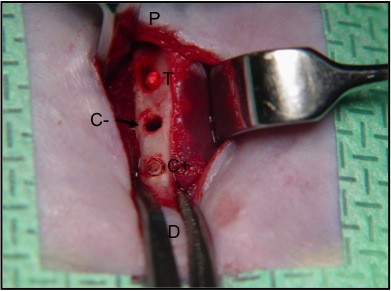
**Intraoperative aspect of the C - cavity (no implantation)**. T and C + cavities are already implanted. The proximal (P) and distal (D) endings are identified.

**Figure 2:**
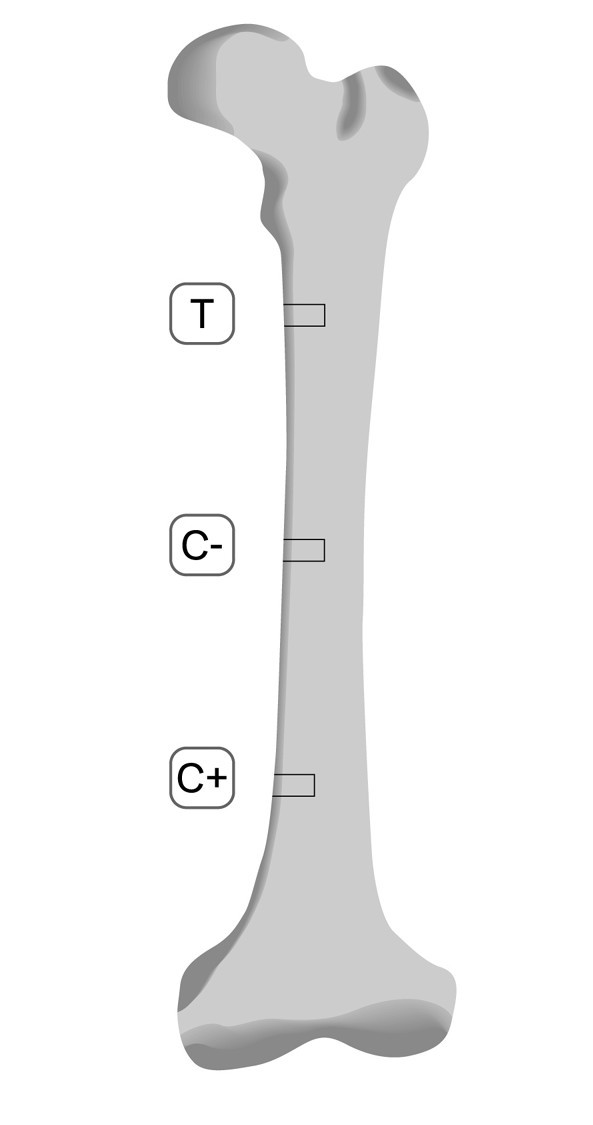
**Test (T), negative control (C - ) and positive control (C + ) cavities on the femur**.

The ostectomized T cavities were filled with a precured block of alpha-TCP. C + cavities received the bone fragment removed from the T hole, whereas the C - cavities were left without any filling (Figures 1 and 2). The wound was sutured in layers (Vicryl - Ethicon, Johnson&Johnson, São José dos Campos, SP, Brazil).

The sample was randomly selected, with two control and one experimental groups. The rats were divided into five groups (n = 6 each), analyzed in different periods after the experimental procedure: three, seven, 14, 21 and 60 postoperative days. The animals were euthanized, and the right hind leg was stored in 10% neutral buffered formalin. The material included in paraffin was prepared with a microtome, following the femoral long axis.

Sections were stained with hematoxylin and eosin, and mounted with Canada balsam. Slides were analyzed with an optical microscope (Model Lambda LQT 2, ATTO Instruments Co., Hong Kong, China), with 40, 100, 250 and 400 magnification. Bone repair was qualitatively evaluated and compared among different groups.

This study is in accordance with the guidelines for animal research established by the State Code for Animal Protection and Normative Rule 04/97 from the Research and Ethics in Health Committee/GPPG/HCPA.

## Results

**Figure 3:**
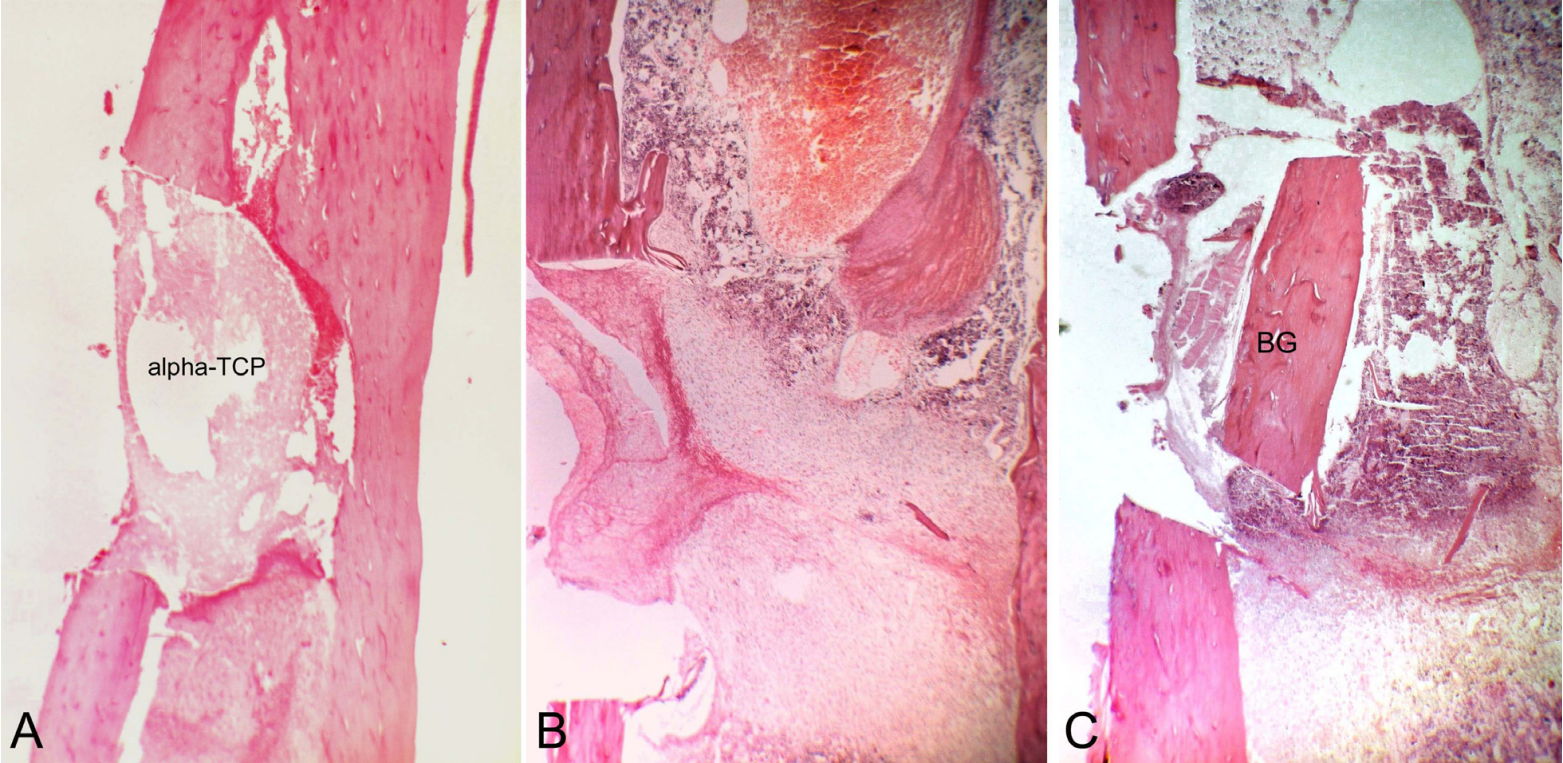
**Histological aspects of samples collected three days after surgery**. A, T cavity, The implanted alpha-tricalcium phosphate (alpha-TCP) can be observed inside the bone cavity. The cortical floor is preserved. (Magnification 40×). B, C - cavity, with no implantation. Important fiberplasia in the region. (Magnification 40×). C, C + cavity, The bone graft is horizontally placed in the cavity. (Magnification 40×).

**Figure 4:**
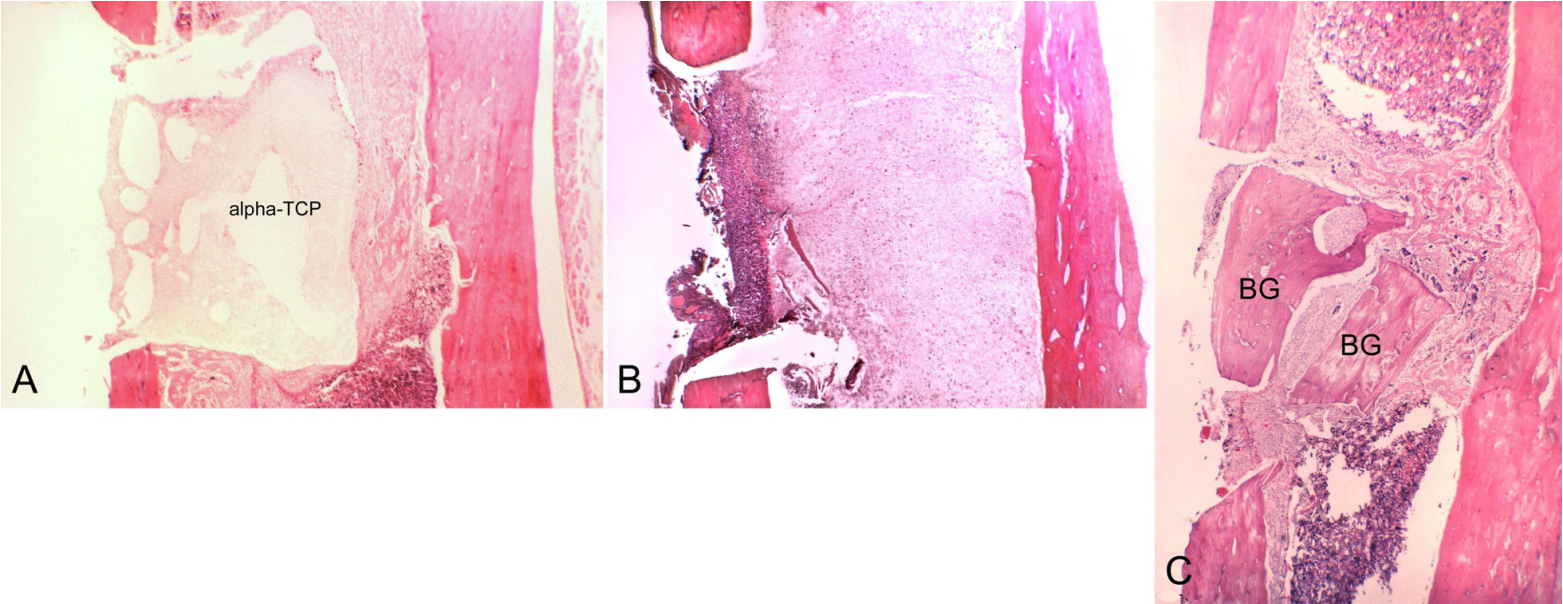
**Histological aspects of samples collected seven days after surgery**. A, T cavity, The stability of the alpha-tricalcium phosphate (alpha-TCP) block implanted in the bone cavity can be observed. (Magnification 40×). B, C - cavity, The implant-free cavity shows import fiberplasia. Bone fragments may be seen in the interior. (Magnification 40×). C, C + cavity, The bone graft (BG) can be seen as two superimposed segments. (Magnification 40×).

**Figure 5:**
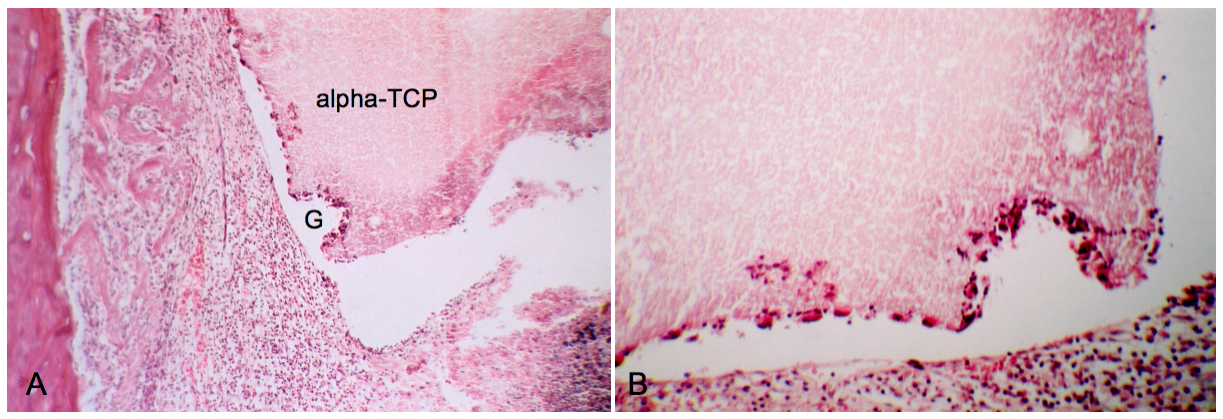
**Histological analysis of the T cavity, in samples collected on postoperative day seven**. A, alpha-tricalcium phosphate (alpha-TCP) block within the cavity. A gap (G) is seen, probably caused by resorption of the material. (Magnification 100×). B, gap in the cement block, caused by activity of the phagocytic system. (Magnification 400×).

**Figure 6:**
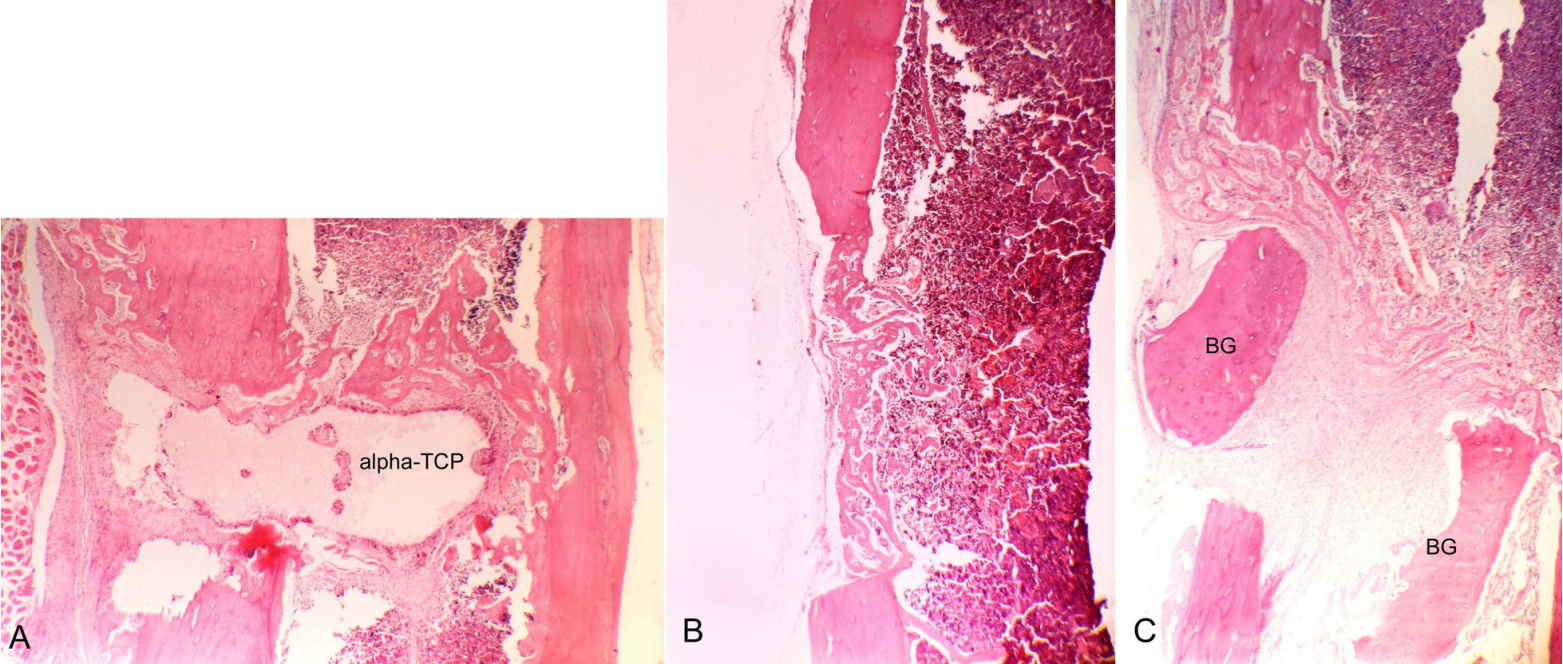
**Histological aspects of samples collected on postoperative day 14**. A, T cavity, Alpha-tricalcium phosphate (alpha-TCP) block implanted in the bone cavity. The material shows irregularities on its surface and interior, evidencing bone neoformation. (Magnification 40×). B, C - cavity, OR In the graft-free cavity, leveling of trabecular bone prevents the closure of the bone wound. (Magnification 40×). C, C + cavity, The grafted segments drift apart, but keep parallel orientation. Extensive fibrosis is observed between the bone grafts. (Magnification 40×).

**Figure 7:**
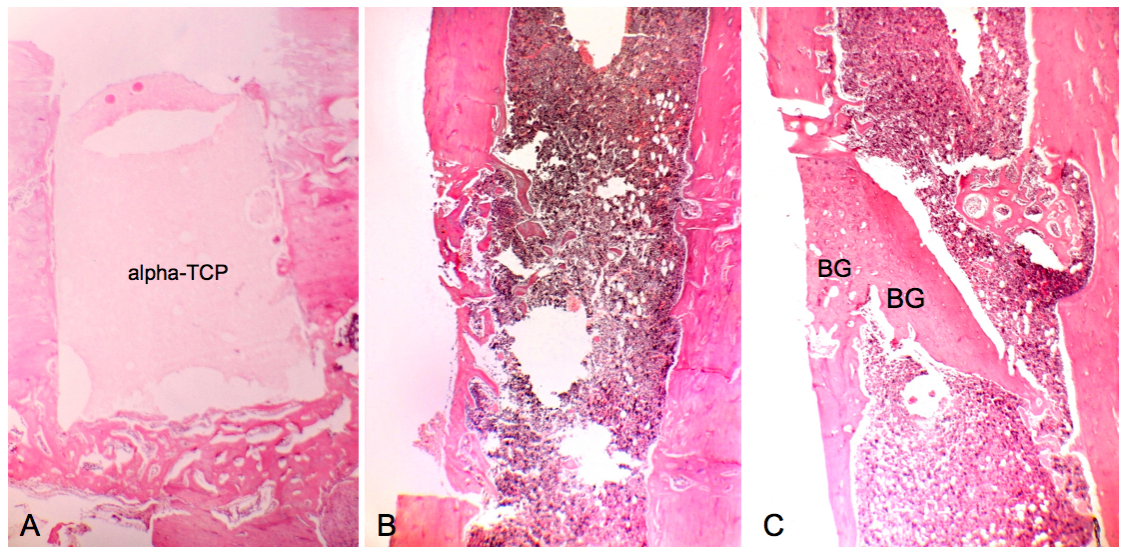
**Histological aspects of samples collected on postoperative day 21**. A, T cavity, The alpha-tricalcium phosphate (alpha-TCP) block occupies all the bone cavity. The regular margins of the cavity contrast with the irregular surface of the material. (Magnification 40×). B, C - cavity, Continued healing of the cortex, in the upper surface of the surgical cavity. The medullary channel shows progressively increasing regularization. (Magnification 40×). C, C + cavity, The bone graft (BG) is seen in continuity with the trabecular area, which is merging into cortical bone. (Magnification 40×).

**Figure 8:**
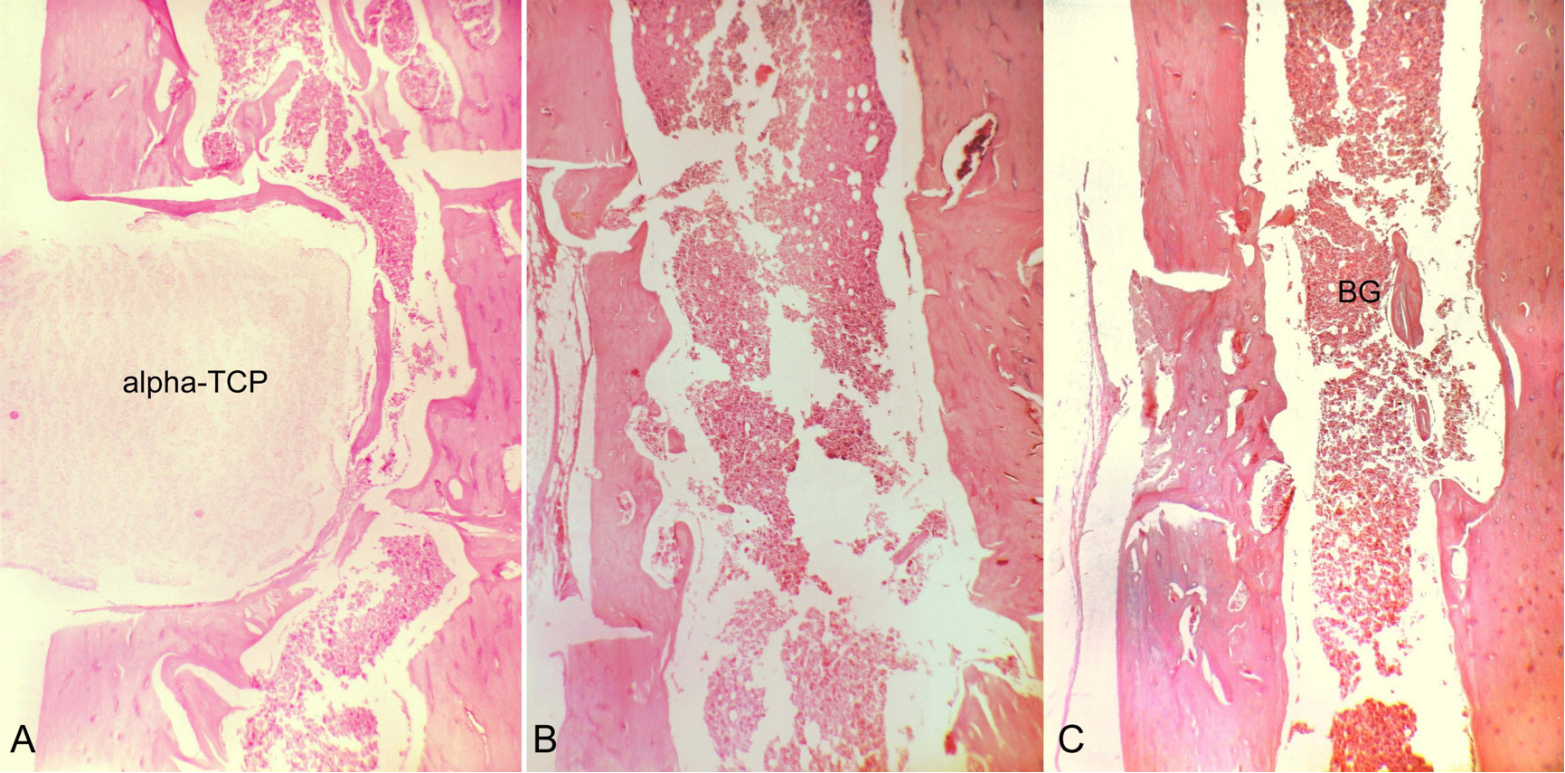
**Histological aspects of samples collected on postoperative day 60**. A, T cavity, The alpha-tricalcium phosphate (alpha-TCP) block is in almost completely surrounded by mature bone tissue. (Magnification 40×). B, C - cavity, Well advanced process of bone healing and remodeling, indicating return to normal of the ostectomized region (Magnification 40×). C, C + cavity, Occlusion of the roof of the ostectomized cavity, with integration of great part of the bone graft (BG) with the cortical bone. (Magnification 40×).

Representative results observed on the different postoperative days are presented in Figures 3, 4, 5, 6, 7 and 8 (days 3, 7, 14, 21 and 60, respectively). Within each group, histological characteristics were described for each of the surgical cavities. To evaluate the process of bone healing, sections were analyzed for the amount of neoformed bone, presence of an inflammatory infiltrate, and reaction against a foreign body.

### T cavity

All samples presented a marked regular interruption of the ostectomized cortical bone, without dislocation of the alpha-TCP block (Figure 3). On postoperative day 3, an intense inflammatory infiltrate, erythrocytes and proliferating fibroblasts were observed, but no bone neoformation (Figure 3). Evident bone neoformation, beginning on the endosteum around the alpha-TCP block, along with granulation tissue characterized by angiogenesis and fiberplasia, were observed on day 7 (Figure 4). Irregularities could be seen on the border of the cement block, with macrophages and giant multinucleated cells (Figure 5).

On postoperative day 14, the neoformation of trabecular bone was evidenced by the reversal (basophil) line, as well as concentric cell areas with bone formation within the block (Figure 6). On day 21, areas of bone tissue in different maturation degrees were observed, with regression of the inflammatory activity and less fibrous tissue in the region. The regularity of the implant-bone surface interface was noteworthy (Figure 7). The marginal bone neoformation was well organized, with bone isthmus invasion and establishment in occasional irregularities of the implanted inorganic structure. On day 60, mature bone tissue replaced the immature tissue. The surface of the alpha-TCP block was invaded by cells, and an outline of neoformed bone surrounded the bone/implant interface (Figure 8).

### C - cavity

A markedly regular interruption of the ostectomized cortical bone was observed in all samples. Three days after the surgery, no bone neoformation was observed. An inflammatory infiltrate and intense fibroblastic proliferation were seen in the medullary space. The fiberplasia process, started in the periosteum, showed invagination towards the cavity (Figure 3). The medullary tissue surrounding the cavity had altered continuity and was rich in megakariocytes. Seven days after surgery, intense fiberplasia was seen, and no bone neoformation (Figure 4). Erythrocytes, moderate angiogenesis and an inflammatory infiltrate were observed in the medullary compartment. On day 14, the samples showed bone neoformation, of a predominantly endosteal nature, blocking the interruption between the cortical areas, and reduced levels of inflammatory infiltrate and fibrous tissue (Figure 6). On day 21, the cortical organization could be seen by the presence of trabecular projections, with maturation of lamellar bone whose thickness was compatible with the original bone structure of the region (Figure 7). The ordered presence of adipocytes in the bone marrow showed that the hematopoietic tissue was mature. On Day 60, there was a small indent in the cortex suggestive of wound healing (Figure 8). The dimension and cellular aspect of the medullary channel returned to normal conditions.

### C + cavity

A markedly regular interruption in the ostectomized cortical bone was observed in all samples. On day 7, accelerated bone neoformation was observed (Figure 4). On day 14, primary bone tissue and osteoblastic cells were seen (Figure 6). The reversal line (basophil line) was also observed between the lamellar and primary bone tissues. The hematopoietic bone marrow showed a tendency towards a normal aspect. On postoperative day 21, the upper surface of the cavity presented bone tissue in different degrees of maturation and osteoblasts which merged its structure with that of the graft surface (Figure 7). On day 60, progressive bone neoformation induced by osteoblasts was seen, as well as bone repair confirmed by the closure of the cortical bone (Figure 8).

## Discussion

The experimental protocol used in this work, established by Puricelli and colleagues [23,24,26,27], has proven very adequate for this type of study. The ostectomized cortical structure showed marked regularity, in all groups and experimental periods investigated. Three and 7 days after surgery, all samples showed the presence of granulation tissue in the cavities, previously described by Junqueira and Carneiro [29] and Burkitt et al. [1].

The biological properties of autogenous bone grafting, considered to be the gold standard [3-5], could be observed seven days after surgery, with an accelerated process of bone neoformation, as compared to the negative control group. The present study also showed that alpha-TCP blocks filled the surgical cavities without the development of inflammatory reactions of significant extension or duration, as already shown in other studies [16,17].

In the present work, we used histological parameters to monitor the process of bone healing. The progress of fracture healing is often difficult to assess, and clinicians have to rely on subjective parameters such as pain or tenderness to palpation to monitor this process. A consistent definition of bone healing is lacking (reviewed by [30]), and many biological markers which are easy to assess on radiographic examination have shown poor correlation with mechanical strength [31]. More recently developed methods, such as micro-computed tomography (micro-CT [33] or structural rigidity analysis [31], have shown potential in monitoring the progression of fracture healing over time. Histological analysis, however, is still considered a valuable tool to asses fracture healing, and has shown good correlation with quantitative methods. In a study aiming to evaluate the role of endothelial progenitor cells on bone regeneration in a rat model, healing was evaluated with radiographic, histological, and micro-CT scans [34]. Histological results, showing that cell-treated animals had significantly higher levels of new bone and vessel formation than controls, correlated with radiographic and micro-CT assessments showing significantly improved parameters of bone volume, density, trabecular number, thickness and spacing, as well as bone surface and bone surface to bone volume ratio for the treated group compared to control.

According to Schenk et al. [7], the ideal biomaterial should show resorption during the remodeling phase, being replaced by bone tissue. These histological results support previous studies by Parker (1995) [19] showing that, simultaneous to bone neoformation in the bone/implant interface, the cement is phagocytosed by macrophages and multinucleated giant cells, adding osteotransductivity to its properties. Cavities filled with the alpha-TCP cement showed, as early as seven days after surgery, accelerated bone neoformation, surrounding the cement blocks. On day 14, concentric cellular areas with bone formation were observed in the interior of the blocks. Similar results were reported by Toquet et al. [21].

In a meta-analysis of histomorphometry and graft healing time of different types of biomaterials used as sinus floor augmentation material in humans, Klijn et al. [35] concluded that autologous bone is still the gold standard. Allogenic, xenogenic or alloplastic graft materials resulted in a significantly lower amount of bone volume as compared to autologous bone grafting. However, a wide variety of scaffolds have shown therapeutic results on the repair of bone defects. Gunatillake and Adhikari [36] reviewed the role of biodegradable synthetic polymers in bone healing, showing their potential in many types of clinical applications. The therapeutic potential of PGA/β-TCP was studied in a rat model [25]. The scaffold presented strong ability for osteogenesis, mineralization and biodegradation for bone replacement.

The alpha-TCP cement formulated by Santos (2002) [22] has the stability property proposed by Shindo et al. [8] as important for biomaterials. Our results showed that, in all groups, bone neoformation involved initially the formation of immature primary bone that was progressively remodeled for production of mature lamellar bone. This process is well known in humans, as described by Burkitt, Young and Heath (1994) [1] and Junqueira and Carneiro (2004) [29]. The design used in the present study did not allow for the investigation of a role for the periosteum in this process. The importance of the periosteum for nutrition of the augmentation area during bone healing has been already described [37]. Due to this activity, which seems to be induced by Bone Morphogenetic Proteins (BMPs) [38], surgeons try to preserve the periosteum while treating bone defects.

Sixty days after surgery, a slight interruption of the ostectomized cortical bone could be seen in C - and C + cavities, whereas in the T cavities there was no occlusion on the cavity roof. As already pointed by Schilling et al. [2], the bone repair process may take months to years to be completed. Resorption of calcium phosphate cements is slow, and the biomaterial may last for up to two years after implantation [20].

The present study evidenced the osteoconductivity property of calcium phosphate cements, that induced vigorous trabecular formation, as already indicated in several reports [6,11,17,18,23].

## Conclusion

The histologic results of the present study show that, in rats, the alpha-tricalcium phosphate [Ca _3_(PO _4_) _2_] developed by Santos (2002) [22] presents the properties of biocompatibility, osteotransductivity and stability. The repair process was initially faster in filled (T and C + ) cavities than in non-implanted (C - ) cavities. The results suggest that the analysis of resorption of this cement should be performed in periods longer than 60 days after surgery.

## Competing interests

The authors declare that they have no competing interests.

## Authors' contributions

EP conceived of the study, participated in its design and coordination. AC carried out the experiments and analyses. DP, GLM and MGL participated in the design of the study and the experimental steps. LAS provided the biomaterial. All authors helped to draft the manuscript and approved its final form.
